# Refined anatomy-based fitting reduces interaural place mismatch and improves speech outcomes, with preliminary evidence for reduced listening effort

**DOI:** 10.1371/journal.pone.0361044

**Published:** 2026-10-26

**Authors:** Anja Kurz, Giovanna Castillo, Kristen Rak, Stephan Hackenberg, Alexander Möltner

**Affiliations:** 1 Department of Oto-Rhino-Laryngology, Head and Neck Surgery, Comprehensive Hearing Center, University of Würzburg, Würzburg, Germany; 2 MED-EL Headquarters, Innsbruck, Austria; LSU Health Shreveport, UNITED STATES OF AMERICA

## Abstract

**Objective:**

This study proposes a refined approach to anatomy-based fitting (rfABF) that aims to reduce interaural place mismatch in bilateral cochlear implant (CI) users with asymmetric electrode array insertions between ears, a condition frequently associated with incomplete cochlear coverage.

**Design:**

Prospective monocenter proof-of-concept study comparing rfABF with conventional clinically based fitting (CBF). ABF maps were prepared using two algorithmically derived methods: one scaling inter-electrode spectral spacing (zipped), and another transposing the ratio between clinical and tonotopic frequencies (transposed). These maps were then refined based on user feedback until a natural sound quality was achieved. Assessments consisted of speech perception in quiet and noise, sound localization, subjective sound quality ratings (HISQUI_19_), and listening effort assessed using pupillometry.

**Study sample:**

Nine experienced adult bilateral CI users with interaural asymmetry in electrode array insertion depth and/or electrode array type.

**Results:**

Bilateral speech understanding improved significantly with rfABF in quiet (CBF: 77.5% ± 8.1%; rfABF: 83.1% ± 8.0%; p = 0.025) and in noise under both S0N0 (signal and noise from front) and S0Ncontra (signal from front, noise from contralateral ear) configurations. Interaural place-of-stimulation mismatch was substantially reduced from a mean of 33.8° ± 27.3° to 6.4° ± 4.2° with rfABF. Exploratory, descriptive pupillometry indicated reduced listening effort in a subset of participants, reflected by lower event-related pupil dilation (ERPD) amplitudes, particularly in cases where speech intelligibility was preserved or improved.

**Conclusions:**

rfABF shows clinical potential to improve speech understanding and facilitate access to binaural cues in bilateral CI users with asymmetric electrode array insertions, with preliminary evidence for reduced listening effort.

## 1 Introduction

Cochlear implant (CI) sound processors assign incoming acoustic frequencies to electrode contacts according to a manufacturer-defined frequency allocation table. Because cochlear duct lengths vary considerably across individuals [1,2] and electrode arrays differ in length and insertion depth, a discrepancy routinely arises between the tonotopic frequency corresponding to a given cochlear location [3,4] and the acoustic frequency that the processor delivers to the electrode contact positioned there. This frequency-to-place mismatch —hereafter termed tonotopic mismatch— has been shown to limit speech perception, prolong post-activation adaptation, and reduce sound quality [1,5,6]. Because the degree of mismatch varies with electrode array design, insertion depth, and individual cochlear anatomy, it is present to some extent in all CI users but is not uniform across devices from any manufacturer [1,7].

Anatomy-based fitting (ABF), also termed tonotopic fitting [5], is an image-guided fitting strategy that seeks to reduce tonotopic mismatch by aligning each electrode contact’s assigned frequency band with the tonotopic frequency corresponding to its actual anatomical position in the cochlea. In clinical practice, postoperative imaging is used to determine each electrode contact’s angular insertion depth (AID). These positions are then processed through otological planning software, which applies a cochlear frequency-position function—typically the Greenwood function [3] —referenced to either the organ of Corti or the spiral ganglion [4], to derive a tonotopic center frequency for each contact. The resulting individualized frequency map replaces the manufacturer’s default logarithmic distribution in the fitting software [8,9]. In current automated ABF implementations, the software exclusively aligns bandpass filters for electrode contacts whose tonotopic frequencies fall within the speech-critical range of 950–3,000 Hz, while lower frequencies are delivered through fine-structure temporal coding strategies where rate coding—rather than tonotopic place alone—plays a dominant role in pitch perception [8]. This automated alignment requires specific spatial prerequisites: a minimum distribution of electrode contacts across the low, mid, and high frequency regions must be met for the software to generate a valid map [10]. The remaining electrode contacts outside the 950–3,000 Hz range are distributed to cover the full audio processor frequency range using a logarithmic approximation, which means that residual mismatch can persist at the frequency boundaries even after ABF is applied [8]. This workflow has been validated across several imaging modalities, including high-resolution flat-panel volume computed tomography [2,11,12], plain X-rays [13,14], and photon-counting CT [15]. Individualized cochlear scaling models have been developed to further refine electrode localization and frequency assignment [16], and recent work has proposed level-adjusted frequency-to-place maps that may provide more physiologically accurate tonotopic references for CI users [17].

A growing body of evidence supports the clinical benefit of ABF in reducing tonotopic mismatch and improving hearing outcomes. In a prospective randomized double-blind crossover study, tonotopic fitting significantly improved speech recognition in noise compared with default fitting in new CI users [18]. ABF has been associated with improved longitudinal speech perception in newly activated CI users [19], as well as enhanced performance over time in experienced users [9]. Beyond speech understanding, this fitting approach yields better perception of musical features [10,20] and improves subjective sound quality for patients undergoing bimodal rehabilitation (Canale et al., 2026). However, these benefits are not universally observed. Several studies have reported comparable speech scores between ABF and default fitting, particularly in experienced users who may have already adapted to their existing maps [8,21], and image-guided fitting approaches explored with other electrode array platforms have yielded mixed results [22]. More broadly, alternative strategies to address tonotopic mismatch have included perimodiolar electrode array designs that reduce the physical distance to neural targets [7]), virtual channel techniques that extend the effective stimulation range beyond the most apical electrode contact [23], and computational approaches that adjust the tonotopic reference itself [17]. The degree of benefit from any mismatch-reduction strategy appears to depend on insertion depth, cochlear coverage, and the magnitude of the baseline mismatch [1,6]. An additional practical challenge is that experienced CI users often resist sudden frequency shifts; the observation that gradual psychoacoustic fine-tuning is needed for successful adoption in this population was described as the “One Click Is Not Enough” challenge by [24].

While ABF as described above addresses tonotopic mismatch within a single ear, bilateral CI users face an additional, distinct problem: interaural place-of-stimulation mismatch. Two types of mismatch must therefore be clearly distinguished. Tonotopic mismatch refers to the discrepancy between the cochlea’s natural frequency map and the frequency assigned to a given electrode contact within a single ear; it is an intraaural phenomenon. Interaural mismatch refers to the difference in cochlear place of stimulation across the two ears for the same acoustic frequency input, arising from differences in electrode array type, insertion depth, or cochlear anatomy between sides [25,26]. Although both types of mismatch frequently coexist, they have distinct perceptual consequences and require different fitting strategies.

The prevalence and magnitude of interaural mismatch in bilateral CI populations have been characterized through large-scale computed tomography analyses [26]. examined 107 bilateral CI users across three manufacturers and found a median interaural insertion-depth mismatch of 23.4° (1.3 mm), with clinically significant mismatch—exceeding approximately 75° or 3 mm—occurring for 13–19% of electrode pairs overall and for at least three electrode pairs in 23–37% of subjects. While average interaural mismatch in bilateral CI populations is modest, these data indicate that a substantial proportion of users have mismatch magnitudes that may degrade spatial hearing.

The consequences of interaural mismatch for binaural processing are well established through foundational psychoacoustic work. The central binaural processor depends on frequency-matched inputs from both ears to resolve binaural cues: interaural mismatch degrades the salience of interaural time and level difference (ITD and ILD) cues in vocoder simulations of bilateral CI listening [27], disrupts binaural image fusion in bilateral CI users [28], and can produce off-center or split auditory images that distort the spatial map. Lateralization using ITDs remains possible with up to approximately 3 mm of interaural mismatch in controlled tasks, but beyond this range binaural fusion deteriorates markedly [28]. Studies comparing interaural electrode pairing methods have further demonstrated that pitch-matched electrode pairs frequently do not correspond to the pairs yielding the best binaural sensitivity, and that CT-based anatomical estimates provide a more reliable basis for optimizing interaural alignment than subjective pitch matching [25,29,30]. A comprehensive review of binaural processing outcomes in bilateral CI users with various forms of asymmetrical hearing has confirmed that interaural asymmetries—whether in insertion depth, hearing history, or speech intelligibility—limit the ability to access binaural benefits including spatial release from masking and binaural redundancy [31].

Critically, while the auditory system shows considerable plasticity for monaural speech adaptation to frequency-shifted allocations, as recently demonstrated in CI recipients who learned to understand speech while alternating between two distinct frequency maps [32], foundational evidence indicates that the brainstem binaural system does not exhibit sufficient plasticity to fully compensate for significant interaural place-of-stimulation mismatches (Bernstein et al., 2021). In that study, binaural and CT-based measures of interaural mismatch were in agreement, whereas pitch-matched electrode pairs deviated from both, suggesting place-pitch plasticity but not binaural-system plasticity. This dissociation provides a strong rationale for clinical correction of interaural mismatch through fitting rather than relying on neural accommodation. Accordingly [26], recommended the interaural alignment of clinical frequency tables for bilateral CI users with insertion-depth mismatch, though the clinical implementation and evaluation of this recommendation had not been addressed.

Kurz et al. demonstrated that ABF can improve bilateral speech outcomes when applied to both ears by using two approaches for frequency alignment: a tonotopic matching approach for deeply inserted arrays (AID ≥ 620°) and a transposition approach for shallower insertions ([24,33]). These studies established that access to the tonotopically correct low-frequency range, requiring an AID of approximately 620°–720° [33], is a prerequisite for direct tonotopic fitting. In a subsequent study of ten bilateral CI users, ABF significantly improved bilateral speech understanding in noise and spatial release from masking compared with conventional clinically based fitting (CBF), demonstrating the clinical feasibility of image-guided bilateral frequency alignment [33]. ABF has also been extended to CI recipients with single-sided deafness, where reducing the mismatch between the CI ear and the normal-hearing contralateral ear improved bilateral speech outcomes in noise [34].

However, in bilateral CI users with incomplete cochlear coverage and pronounced interaural asymmetry, standard ABF applied independently to each ear may not suffice to align interaural stimulation. When electrode arrays do not reach the apical cochlear regions, low-frequency inputs must be assigned to more basal electrodes, resulting in compressed or distorted frequency-place relationships within each ear [34]. In bilateral recipients with asymmetric insertions—arising from differences in electrode array length, surgical approach, or cochlear anatomy—the resulting interaural place-of-stimulation offsets can exceed the binaural tolerance range [26,28]. Reducing tonotopic mismatch within each ear independently does not guarantee that the same acoustic frequency will stimulate corresponding cochlear locations in the two ears [25].

The present study addresses this gap with a refined ABF (rfABF) approach that explicitly targets interaural place-of-stimulation mismatch. Rather than fitting each ear independently to its own tonotopic map, rfABF uses the frequency allocation of the participant’s preferred ear as an anchor and adjusts the contralateral ear’s frequency map to minimize the interaural place-of-stimulation difference between the two sides. Two algorithmic methods are employed to calculate the contralateral frequency allocation: one that preserves the inter-electrode spectral spacing of the reference map and scales it proportionally to the interaural tonotopic differences (hereafter termed the zipped method), and another that maintains the ratio between the clinical frequency allocation and the tonotopic electrode frequency on the reference side and applies this ratio to the contralateral side (hereafter termed the transposed method). In both cases, the calculated maps are further refined through psychoacoustic fine-tuning based on participant feedback, limited to adjustments of most comfortable levels without modification of frequency boundaries or center frequencies, so as to preserve the algorithmically defined place alignment and isolate interaural matching effects. This approach builds on the methodology and clinical guidance developed in the preceding bilateral ABF work [24,33] and extends it specifically to cases with incomplete cochlear coverage and pronounced interaural asymmetry.

Beyond speech performance, reducing interaural mismatch may also decrease listening effort. CI users often experience elevated listening effort when integrating degraded or mismatched auditory signals, and this effort—though not captured by standard speech intelligibility scores [35] —has meaningful implications for fatigue, quality of life, and everyday communication [36]. Interaural asymmetry in bilateral CI users has been associated with increased listening effort, with bilateral listening sometimes proving more effortful than better-ear listening alone when interaural inputs are poorly matched [37,38]. Pupillometry—the measurement of task-evoked changes in pupil diameter—is a well-validated objective index of listening effort in hearing research [39,40]. Pupil dilation reflects the engagement of cognitive resources during demanding listening tasks and is sensitive to spectral degradation of the type produced by tonotopic mismatch [41,42]. Importantly, the relationship between task difficulty and pupil dilation is non-monotonic: at very high difficulty levels, listeners may disengage, producing reduced rather than increased dilation [35,43], a pattern that must be considered when interpreting individual variability in effort responses.

The present study compared rfABF with conventional CBF in nine bilateral CI users with incomplete cochlear coverage and asymmetric electrode array insertions between ears. We assessed (1) the reduction in interaural place-of-stimulation mismatch achieved by rfABF; (2) bilateral speech understanding in quiet and noise; (3) sound localization and self-perceived sound quality; and (4) listening effort as indexed by task-evoked pupil dilation during speech-in-noise processing. We hypothesized that rfABF would reduce interaural mismatch and improve bilateral speech outcomes, and explored whether these changes would be accompanied by reduced listening effort.

## 2 Materials and methods

### 2.1 Study design and participants

This prospective experimental study was approved by the Ethics Committee at the Medical University of Würzburg (ethics approval number: 204/20) and conducted in accordance with the Declaration of Helsinki. The recruitment period started on 15.09.2024 and ended on 25.05.2025. All participants provided verbal and written informed consent prior to study start.

To participate in the study, candidates had to meet all of the following inclusion criteria: 1) have an available post-operative flat panel volume computed tomography (fpVCT) (Siemens AG, Forchheim, Germany) image with a secondary reconstruction of 99 µm, depicting incomplete cochlear coverage; 2) be at least 18 years old at study start; 3) have post-lingual bilateral severe-to-profound hearing loss; 4) be a bilateral CI user with at least 6 months of experience with the SONNET 2 or RONDO 3 audio processor (both MED-EL, Innsbruck, Austria); 5) have at least 10 active electrode contacts on each side; 6) use the FSP, FS4, or FS4-p sound coding strategy on each side; 7) have a speech understanding with each CI of ≥ 25% on a monosyllabic word test at 65 dB sound pressure level (SPL), or a speech reception threshold (SRT) of ≤ 20 dB signal-to-noise ratio (SNR) on a sentence-in-noise test; 8) be willing and able to provide feedback on the fitting map and process; and 9) give their signed and dated informed consent before participating in any study-related procedures. Candidates who did not fulfill the inclusion criteria were excluded.

This was a prospective proof-of-concept study. Given the limited pool of bilateral CI users meeting the inclusion criteria, the sample size was necessarily small, and the study was designed to establish feasibility and generate hypotheses rather than to provide definitive confirmatory evidence.

### 2.2 Electrode localization and baseline maps

Electrode contact positions were extracted from fpVCT images using clinical surgical planning software (OTOPLAN® version 4, CASCINATION AG, Bern, Switzerland). This information was subsequently imported into clinical fitting software (MAESTRO version 9.0.5, MED-EL, Innsbruck, Austria), where the positions of individual electrode contacts were displayed within the manufacturer’s pre-set frequency band distribution (range 70–8500 Hz) of the audio processors.

For each participant, tonotopic electrode contact frequencies OC(i) were computed in OTOPLAN using the Greenwood function [3,4] applied to each electrode’s anatomical position within the cochlea. The index i (i=1,…,12) enumerates electrode contacts from the most apical (i=1) to the most basal (i=12) and, by convention, also denotes the corresponding filter band in the MAESTRO map. In MAESTRO, each filter band represents the range of acoustic frequencies assigned to a single electrode contact during sound coding. It is defined by a lower and an upper cutoff frequency and by a center frequency. Each electrode contact is therefore linked to 2 complementary frequency values: the tonotopic electrode frequency OC(i) derived from its anatomical location and the center frequency CBF(i) of its corresponding filter band in the CBF map.

Each participant selected their preferred ear based on subjective perception of hearing. This side is referred to as the “reference ear”. The opposite side is referred to as the “contralateral ear”.

For each index i, the tonotopic electrode contact frequency OC(i) and the corresponding filter band center frequency CBF(i) were denoted ${OC}_{ref}(i)$ and ${CBF}_{ref}(i)$ for the reference ear, and ${OC}_{contra}(i)$ and ${CBF}_{contra}(i)$ for the contralateral ear. The CBF frequency maps of the reference and contralateral ears are given by the ordered sets $\left\{{CBF}_{ref}(i)\right\}$ and $\left\{{CBF}_{contra}(i)\right\}$, defined for i=1 (apical)… i=12 (basal). In the following sections, we refer to these clinically based maps simply as ${CBF}_{ref}$ and ${CBF}_{contra}$.

For both ears, most comfortable levels (MCLs) were assessed for each filter band and adjusted as needed using standard clinical procedures.

### 2.3 Refined anatomy-based fitting (rfABF)

For each participant, the reference ear retained its CBF frequency map $\left({CBF}_{ref}\right)$, corresponding to the participant’s habitual fitting program used in daily listening, while the rfABF map was applied exclusively to the contralateral ear.

Two approaches were used to generate ABF maps: zipped ABF and transposed ABF. These initial configurations are referred to as “calculated ABF” maps. For each participant, both calculated ABF maps (zipped ABF and transposed ABF) were created and evaluated.

To select between zipped ABF and transposed ABF, both maps were activated sequentially in MAESTRO using the live stimulation mode, which allows real-time auditory input through the audio processor during the fitting session. While in MAESTRO live mode, participants engaged in a short conversational interaction under each fitting condition. Based on this immediate real-time listening experience, participants made an acute subjective decision regarding which of the two ABF maps provided the more natural and preferred sound quality. This participant-selected ABF map was then retained for further refinement.

For the selected ABF approach (zipped ABF or transposed ABF), the calculated center frequency values (see below) were entered into the MAESTRO fitting software with “Link Frequency Bands” disabled. After all center frequencies were entered, the Link Frequency Bands were re-enabled, allowing MAESTRO to automatically recalculate the lower and upper cutoff frequencies of each band to ensure continuity between adjacent bands.

Psychoacoustic fine-tuning was then performed based on user feedback and was limited to adjustments of most comfortable levels (MCLs) across filter bands, without modification of frequency boundaries or center frequencies. This process resulted in a single rfABF map for each participant.

#### 2.3.1 Zipped ABF.

The zipped ABF method preserves the spectral spacing between adjacent electrode contacts in the reference map and transfers this spacing to the contralateral map.

For each pair of consecutive indices i and i+1 on the reference ear, the differences between the tonotopic electrode contact frequencies and the filter-band center frequencies were calculated as


$$\begin{array}{c} \Delta {CBF}_{ref}(i)={CBF}_{ref}(i+\text{1})-{CBF}_{ref}(i) \end{array}$$ (1)

$$\begin{array}{c} \Delta {OC}_{ref}(i)={OC}_{ref}(i+\text{1})-{OC}_{ref}(itext. \end{array}$$ (2)

The tonotopic frequency difference between the sides for the same electrode contact index i was then calculated as


$$\begin{array}{c} \Delta {OC}_{\;ref\;vs.\;contra}(i)={OC}_{contra}(i)-{OC}_{ref}(itext. \end{array}$$ (3)

A scaling factor $F_{i}$ was computed as the ratio between the tonotopic frequency difference across sides and the tonotopic spacing between consecutive electrode contacts on the reference ear


$$\begin{array}{c} F_{i}=\frac{\Delta {OC}_{\;ref\;vs.\;contra}(i)}{\Delta {OC}_{ref}(i)}\text{.} \end{array}$$ (4)

Using this factor (4), the center frequency of the filter band corresponding to index i on the contralateral ear was calculated as


$$\begin{array}{c} {ABF}_{calculated}(i)={CBF}_{ref}(i)+F_{i}*\Delta {CBF}_{ref}(itext. \end{array}$$ (5)

For the most basal electrode contact (i=12), where no adjacent reference-ear contact (i+1 = 13) exists, the zipped ABF formulation converges to the proportional coefficient method, yielding the same center-frequency estimate as the transposed ABF approach described below.

#### 2.3.2 Transposed ABF.

The transposed ABF map was first described by Kurz et al. for bilateral CI users who did not tolerate a strictly tonotopic fitting (Kurz et al., 2023). In this approach, the ratio between the tonotopic electrode contact frequencies and the filter band center frequencies of the reference ear is first calculated as


$$\begin{array}{c} F_{i}=\frac{{CBF}_{ref}(i)}{{OC}_{ref}(i)}\text{.} \end{array}$$ (6)

The same ratio is then applied to the tonotopic electrode contact frequencies of the contralateral ear to determine the center frequency of each filter band:


$$\begin{array}{c} {ABF}_{calculated}(i)={OC}_{contra}(i)*F_{i}. \end{array}$$ (7)

This proportional transformation maintains the same tonotopic relationship on both sides, ensuring consistent relative positioning between clinical filter-band center frequencies and tonotopic electrode contact frequencies without enforcing absolute Greenwood alignment.

### 2.4 Apparatus and experimental setup

All assessments were conducted in a sound-attenuated room. Nine identical active loudspeakers (M52 Klein & Hummel, Georg Neumann GmbH, Berlin, Germany) were positioned at 22.5° intervals across the frontal horizontal plane, covering 180° (S1 Fig, Supplemental Digital Content). Each loudspeaker was positioned at a distance of 1.5 meters from the listener, who was seated at the center of the array. Multichannel digital-to-analog conversion was facilitated by a Focusrite Scarlett 18i20 (2nd Generation, High Wycombe, UK). Custom programs developed in MATLAB (MathWorks, Natick, MA, USA) were used to conduct speech understanding tests, sound localization, and pupillometry.

For the core speech-understanding battery, auditory stimuli were presented from loudspeakers positioned at −90°, 0°, and +90° azimuth, corresponding to positions 1, 5, and 9 in S1 Fig (numbered left to right; Supplemental Digital Content).

An additional speech-in-noise test was conducted for the collection of pupillometry data. A computer screen was positioned precisely between loudspeakers 3 and 4, located at −11.25° and +11.25° azimuth relative to the participant (S1 Fig, Supplemental Digital Content). For this task, participants were asked to shift their body slightly to the left to face the screen, while auditory stimuli were presented from loudspeakers 3 and 4. Pupil diameter was measured at a frequency of 120 Hz using a Pupil Core eye-tracking headset (Pupil Labs GmbH, Berlin, Germany) during the speech-in-noise block. No pupillometry data were collected during the subsequent speech-understanding tasks. The experimenter monitored signal quality in real time.

### 2.5 Procedure and conditions

#### 2.5.1 Overall procedure.

Participants used their own audio processors during testing. Prior to the commencement of the test, a thorough inspection of the audio processor was conducted to ascertain its operational integrity, encompassing a comprehensive evaluation of the microphone. Both audio processor models (SONNET 2 and RONDO 3) share an identical technology platform and front-end signal processing architecture, ensuring technology-level parity across participants. Assessments were conducted at 2 time points: baseline and follow-up (≥ 4 weeks post-baseline). At baseline, participants were tested with their accustomed CBF map. Subsequently, 2 anatomy-based fitting maps (zipping ABF and transposed ABF) were calculated for each participant and evaluated sequentially during the fitting session, as described in Section 2.3.1. Participants selected their preferred ABF map in terms of sound naturalness, which was then further refined based on psychoacoustic fine-tuning. They were then refitted using this participant-selected rfABF map (zipped ABF or transposed ABF) and instructed to use only the rfABF map until follow-up. At ≥ 4-week follow-up visit, all tests were repeated using the same measures and procedures. At this visit, processor data-logging (SONNET 2/RONDO 3) was reviewed, and we confirmed that participants used the rfABF map for a mean of more than 12h/day per subject. At both visits, participants were tested with both the CBF and the rfABF map; the pupillometry results reported here are those obtained at the follow-up visit.

#### 2.5.2 Speech perception assessment.

##### 2.5.2.1 Speech understanding in quiet:

Speech understanding in quiet was assessed using the German Freiburg Monosyllables test at 65 dB SPL with the speech signal coming from the front (S0). Testing was conducted in 3 setups: unilaterally with only the reference-ear CI (hereafter referred to as “reference”), unilaterally with only the contralateral-ear CI (hereafter referred to as “contralateral”), and bilaterally. Scores are reported as the percentage of correctly identified words.

##### 2.5.2.2 Speech understanding in noise:

Speech understanding in noise was assessed using the Oldenburg Sentence Test (OLSA) with a variable masker level and a constant speech level of 65 dB SPL. The OLSA test uses an adaptive test procedure to estimate the SNR required for 50% correct words recognition. To minimize training and fatigue effects, list and condition order were randomized. Testing was conducted in two spatial configurations: 1) S0N0, with speech and noise from the front, and 2) S0Ncontralateral, with speech from the front and noise from the contralateral ear.

##### 2.5.2.3 Self-perceived sound quality

Participants rated perceived sound quality using the Hearing Implant Sound Quality Index (HISQUI_19_) [44]. This questionnaire consists of 19 items, each rated on a scale from “Always” (7 points) to “Never” (1 point), and includes a “Not applicable” option (0 points). The total score is the sum of all item scores and is interpreted as follows: < 30 “very poor”, 30–60 “poor”, 61–90 “moderate”, 91–110 “good”, and >111 “very good” sound quality.

##### 2.5.2.4 Sound localization assessment

Sound localization was assessed using the setup described in Section 2.4 (see S1 Fig, Supplemental Digital Content). Auditory stimuli consisted of a broadband CCITT signal and a CCITT noise filtered with a head-related transfer function (HRTF). To minimize the use of non-spatial cues, stimulus type (CCITT or HRTF-filtered CCITT noise) and sound pressure level (SPL) were roved across trials, with levels of 65-, 70-, and 75-dB SPL.

In each trial, a single stimulus was presented from one randomly selected loudspeaker. Participants verbally reported the number of the loudspeaker that best matched the perceived direction of the source, and responses were recorded by the experimenter.

Each loudspeaker position was tested 18 times (2 stimulus types x 3 presentation levels x 3 repetitions), resulting in a total of 162 stimulus presentations across the 9 loudspeakers. Stimulus presentation order was fully randomized across the entire session.

Localization performance was quantified using root-mean-square (RMS) error to assess overall localization accuracy, signed bias to evaluate systematic lateralization, and standard deviation (SD) of the angular response error to characterize response variability.

##### 2.5.2.5 Speech-in-noise pupillometry assessment

Pupillometry was conducted using the setup described in Section 2.4 (see S1 Fig, Supplemental Digital Content). Participants sat at the array center and oriented slightly leftward to face the screen at approximately 1.8 meters viewing distance. The screen displayed a medium-dark grey background throughout to stabilize luminance.

Before testing, participants rested for 5 minutes in the room to adapt to the lighting. The bilateral S0N0 speech-reception threshold (SRT; 50% correct) was first determined as described in Section 2.5.2.2. For pupillometry, the test SNR was set to SRT + 5dB. Within each testing visit, the exact same SNR was applied to both the CBF and rfABF maps, ensuring that map comparisons were conducted at an identical physical acoustic SNR. Because the SRT was re-determined at each visit, the applied SNR could differ between visits.

Pupil diameter was recorded continuously and time-locked to sentence presentation. Each trial began with a 1-second baseline during which participants viewed a blank screen. A central fixation marker then appeared and remained visible for the remainder of the trial. The background noise was gated, beginning 1 second before target sentence onset. The target OLSA sentence began at t = 2 s and lasted approximately 5 seconds (sentence dependent). After sentence offset, the noise continued for an additional 2 seconds. At approximately t = 7 seconds, the fixation marker changed prompting participants to respond verbally. Participants repeated the sentence aloud, filling in missing or unclear words when necessary. All responses were documented for later analysis. Responses were recorded by the experimenter, who then advanced to the next trial. Short pauses were provided between sentence lists. Room lighting and screen luminance conditions were kept constant across participants.

### 2.6 Pupillometry processing and data cleaning

Continuous pupil diameter (mm) was extracted for each trial. Trials were preprocessed and analyzed with custom MATLAB scripts (version 2024a, The MathWorks Inc., Natick, MA). For each participant, data from a single eye were used, given the high correlation between eyes [45].

Within each trial, samples deviating more than 3 standard deviation (SD) below the trial mean trace were classified as blinks or brief signal losses (e.g., head motion). Trials with > 20% blinks/missing samples were excluded. In the remaining trials, identified gaps were linearly interpolated using the preceding 5 and the following 8 samples. Finally, a 5-point moving average filter was then applied to attenuate high frequency artifacts.

Event-related pupil dilation (ERPD) was expressed as percent change relative to baseline, calculated per trial as %ERPD = 100 x (observation – baseline)/baseline, where “baseline” was the 1-second interval immediately preceding noise onset (defined as time 0 in all figures).

For each participant and condition, grand means were obtained by averaging baseline-corrected ERPD across trials. Mean ERPD was then extracted in 3 a priori time-windows: 1) “Background” (0.5–3.5 s), capturing the response to the ongoing noise and the initial target-word processing response; 2) “Peak” (3.5–5.5 s), capturing the phasic dilation to the target sentence; and 3) “Retention” (5.5–7 s), capturing the return toward baseline after peak dilation. Peak latency was defined as the time of maximal dilation within the peak window for each trial.

### 2.7 Statistical analysis methods

#### 2.7.1 Behavioral data and speech outcomes.

Participant characteristics and outcome measures are reported as mean ± SD unless stated otherwise. Data normality was confirmed using the Kolmogorov-Smirnov and Shapiro-Wilk tests. Paired sample t-tests were conducted to assess differences between the CBF map and the rfABF map across test conditions (i.e., reference ear, contralateral ear, bilateral). The significance level was set to α ≤ 0.05. To address multiple comparisons within each outcome measure, p-values were adjusted using the Holm-Bonferroni method. Because the sample size was fixed by participant availability, a sensitivity power analysis was performed to determine the smallest effect detectable under the study design rather than a post-hoc power calculation, which is uninformative when computed from observed effects. For a two-tailed paired t-test with n = 9, α= 0.05, and 80% power, the minimum detectable effect size was $d_{z}\;\approx \;\text{1}.\text{0}\text{7}$ ($d_{z}\;\approx \;\text{1}.\text{1}\text{6}$ for pupillometry subset of n=8). The design was therefore adequately powered only to detect large within-subject effects; non-significant comparisons should be interpreted as inconclusive rather than as evidence of no effect.

All statistical analyses were performed using IBM SPSS Statistics Version 25 (IBM, Armonk, New York, USA). Figures were generated using GraphPad Prism (Version 8.1, GraphPad Software, San Diego, USA).

#### 2.7.2 Interaural place mismatch model.

Interaural place mismatch (Contralateral – Reference, in degrees) was analyzed using a linear mixed-effects (LME) model, following the approach of [25]. For each participant and fitting method (CBF and rfABF), interaural place mismatch was evaluated at reference-ear filter-band center frequencies ${CBF}_{ref}(i)$ by comparing the angular electrode contact position in the contralateral ear to that of the reference ear at matched frequency allocations. All electrode contacts indexed by i with available center-frequency information and corresponding angular insertion-depth estimates on both sides were considered for analysis. Electrode contacts were included when a reference-ear center frequency ${CBF}_{ref}(i)$ was defined and the contralateral angular insertion depth could be obtained at this reference-ear frequency, yielding a defined interaural place mismatch value.

All electrode contact-level observations meeting these criteria were pooled into a long-format dataset (N=186), containing SubjectID, fitting Method (CBF vs. rfABF) log-transformed reference-ear center frequency (in kHz), and signed interaural place mismatch (MismatchDeg).

An LME model was then fitted with signed interaural place mismatch as the dependent variable, fitting Method, log-transformed reference-ear center frequency, and their interaction as fixed effects, and a random intercept for Subject ID to account for repeated measurements across electrode contacts within participants. This specification mirrors Bernstein et al. (2021) and allows direct assessment of systematic differences in interaural mismatch between CBF and rfABF across the frequency range.

Models were fit in MATLAB R2024a (Statistics and Machine Learning Toolbox, The MathWorks Inc., Natick, Massachusetts, USA) using fitlme function. Fixed effects were evaluated using Satterthwaite’s approximation for denominator degrees of freedom and are reported as F statistics with associated p-values. While [25] reported χ² statistics derived from likelihood-ratio testing; the present different inferential framework yields directly comparable fixed-effect patterns.

In addition to the electrode contact-level LME analysis, subject-level summaries were computed to provide an overall measure of mismatch magnitude. For each participant and fitting method, the absolute interaural place mismatch was averaged across electrode contacts. These mean absolute mismatch values were compared between CBF and rfABF using a paired t-test. Given the small sample size, a Wilcoxon signed-rank test was additionally performed as a sensitivity analysis to assess the robustness of the paired comparison to distributional assumptions; both approaches yielded consistent results. Effect size for the paired comparison was quantified using Cohen’s dz.

#### 2.7.3 Bootstrap confidence intervals.

Uncertainty for localization outcomes (RMS error and signed bias) was quantified using nonparametric bootstrap at both the participant and group levels. At the participant level, trials were resampled with replacement with each participant and map (CBF, rfABF) across 10000 iterations; RMS error and signed bias were recomputed on each iteration, and the 2.5^th^ and 97.5^th^ percentiles of the resulting distributions defined participant-level 95% percentile intervals. At the group level, uncertainty for the mean localization metric for each map was obtained via a subject-level bootstrap: participants were resampled with replacement over 10000 iterations, carrying forward each participant’s point estimate for the map. The 2.5^th^ and 97.5^th^ percentiles of the bootstrap distribution of the mean defined the two-sided 95% percentile intervals.

## 3 Results

### 3.1 Participants

Nine participants were included in the study. Their mean age at the time of testing was 59.6 years (± 7.4 years) and their mean duration of CI use was 3.6 years (± 3.2 years) (Table 1).

**Table 1:** Participant demographics and device characteristics.

Participants	Age (years)	Implanted side	CI use (years)	Implant	Electrode Array	E1 Angular Insertion depth	A- Value	H- value	B-value	CDL
In mm	In mm	In mm	In mm
ID01	52	Right	3	Mi12XX	FLEX28	583.9°	*9.1*	*4.3*	*6.5*	34.7
		Left^**‡**^	2	Mi12XX	FLEX28	554.6°	*9.0*	*4.2*	*6.7*	35.4
ID02	64	Right^**‡**^	5	Mi12XX	FLEX28	599.4°	9.2	4.2	6.4	34.2
		Left	12	SONATA	Standard	584.7°	9.7	3.9	6.8	36.6
ID03	66	Right^**‡**^	3	Mi12XX	FLEX28	535.4°	9.0	4.0	6.9	36.1
		Left	1	Mi12XX	FLEX28	418.9°	9.6	4.1	7.3	38.3
ID04	58	Right	1	Mi12XX	FLEXSOFT	569.4°	9.4	4.0	7.3	38.1
		Left^**‡**^	1	Mi10XX	FLEXSOFT	586.0°	9.0	4.2	7.0	36.6
ID05	66	Right	1	Mi12XX	FLEX28	647.1°	9.2	3.6	7.0	36.9
		Left^**‡**^	6	Mi12XX	FLEX28	532.4°	9.1	4.0	7.2	37.2
ID06	47	Right	5	Mi12XX	FLEX24	397.7°	9.0	4.4	7.0	36.4
		Left^**‡**^	6	Mi12XX	FLEX24	417.7°	8.9	4.4	7.0	36.1
ID07	51	Right^**‡**^	5	Mi12XX	FLEX28	512.8°	8.9	3.7	7.3	37.4
		Left	1	Mi12XX	FLEX28	579.6°	9.1	3.8	7.4	37.9
ID08	69	Right	1	Mi12XX	Standard	576.4°	9.5	4.0	6.7	36.0
		Left^**‡**^	3	Mi12XX	Standard	655.3°	9.2	4.1	7.0	36.7
ID09	62	Right	1	Mi12XX	FLEX28	557.8°	9.6	3.5	7.7	39.9
		Left^**‡**^	3	Mi12XX	FLEX28	518.7°	9.7	3.7	7.7	39.8

*Note.* IDs are alphanumeric. **Age (years)** is age at test; **CI use (years)** is time since implantation. **Implant type:** Mi12XX = SYNCHRONY series; SONATA and Mi10XX (CONCERTO) = alternate series. **Electrode array lengths:** STANDARD and FLEXSOFT = 31.5 mm; FLEX28 = 28 mm; FLEX24 = 24 mm. **AID** = angular insertion depth of the most apical electrode (E1), reported in degrees. **‡Reference ear** denotes the ear subjectively perceived as the better-hearing ear.

### 3.2 Individual fitting profiles at baseline

Filter-band center frequencies assigned to individual electrode contacts (shown on a logarithmic scale) were plotted against the AID for each participant, with CBF shown for both ears and rfABF for the contralateral ear (Fig 1A). Tonotopic frequency-angle reference curves for the organ of Corti and spiral ganglion, derived from Greenwood functions in OTOPLAN, are shown for anatomical comparison. Clear divergences between contralateral CBF and rfABF mappings are evident in participants 1, 4, 5, 8, and 9. In participant 5, the contralateral array achieves a deep insertion (AID ≈ 647.1°), yielding a clinical fit that closely follows the Greenwood function. A similar observation applies to participant 8, in whom the reference ear was inserted to a greater degree of depth (655.3°), allowing for improved approximation.

**Fig 1:**
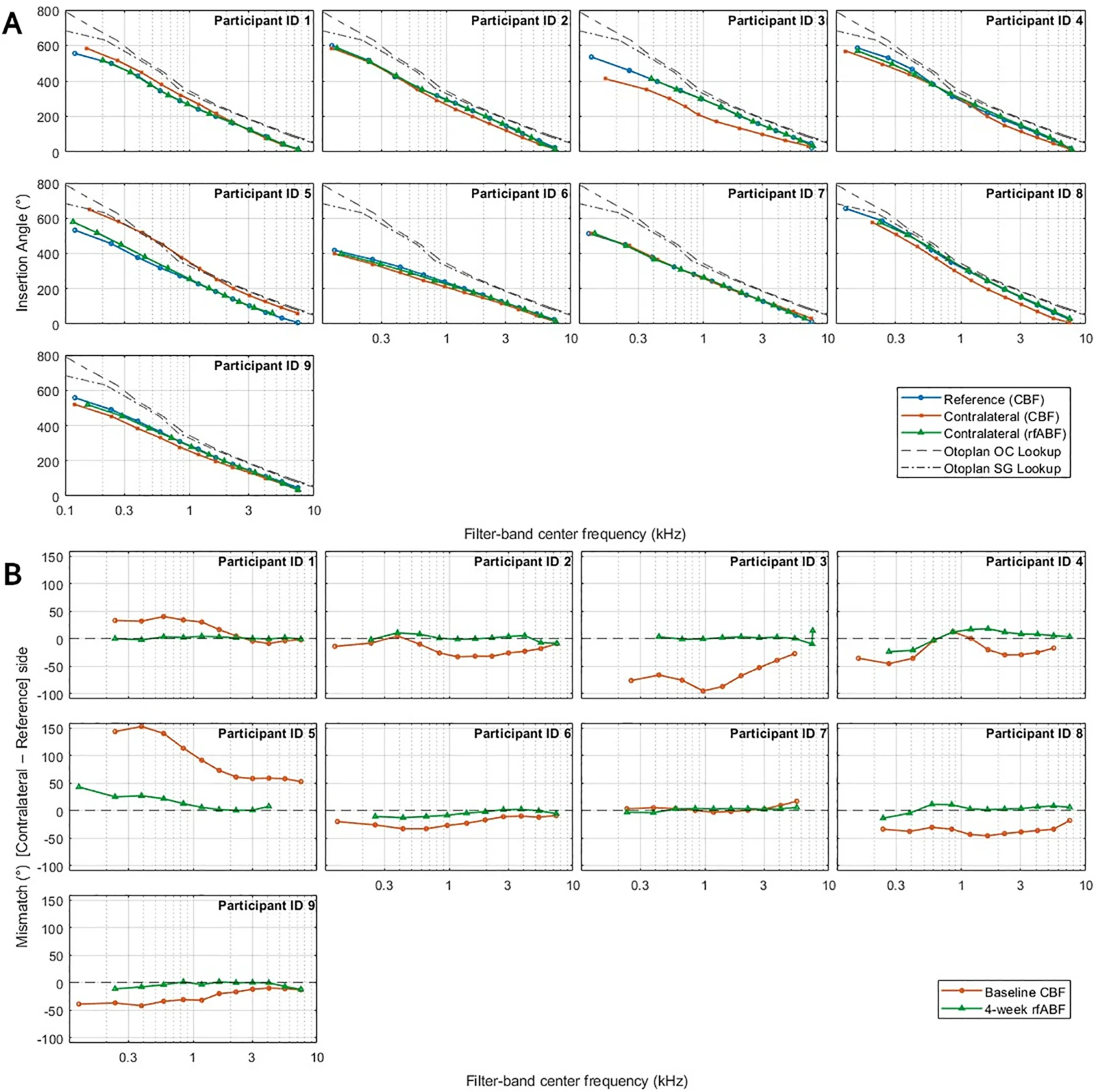
Electrode contact frequency-place profiles and interaural place mismatch by participant. **A**. For each participant (IDs 1–9), angular electrode contact position (°) is plotted against the filter-band center frequency (kHz; log scale 0.1-10). Curves show the reference ear with the clinical baseline fit (CBF; blue), the contralateral ear with CBF (red), and the contralateral ear with the refined anatomy-based fit (rfABF; green). Dashed lines depict manufacturer lookup functions derived from OTOPLAN for organ of Corti (OC; grey, dashfigued) and spiral ganglion (SG; grey, dashed-dot). **B**. Interaural place mismatch (Contralateral – Reference, in degrees) computed at matched reference-side filter-band center frequencies for the same participants. Red circles show mismatch with contralateral CBF; green triangles show mismatch with the contralateral rfABF. CBF = clinically based fitting; rfABF = refined anatomy-based fitting.

### 3.3 Interaural place mismatch

In the present study, interaural place mismatch refers to interaural place-of-stimulation. Electrode contact positions remain fixed inside each cochlea, thus the interaural place mismatch arises because the same acoustic frequency stimulates different cochlear locations between ears due to differences in frequency allocation.

Interaural place mismatch was calculated for each participant at baseline and at 4 weeks post-rfABF as the signed difference in angular electrode contact position between the contralateral and reference ears evaluated at matched reference-side filter-band center frequencies (Contralateral – Reference, in degrees; Fig 1B). Specifically, for each reference-side filter band, the angular insertion depth corresponding to its center frequency was identified on both sides, and interaural place mismatch was defined as the angular difference between the contralateral and reference ears at that frequency location. At baseline, mismatch was derived from CBF maps in both ears. At 4 weeks, it was calculated between the reference ear using the CBF map and the contralateral ear using the rfABF map.

For all participants, the rfABF approach substantially reduced the magnitude of interaural mismatch, reaching up to ~150° in participant 5 and ~100° in participant 3 for the CBF map. For most participants (IDs 1, 2, 4, 6, 8, 9) the interaural mismatch was approximately 50°. One participant (ID 7) showed only a small mismatch ~25°, requiring minimal correction.

ABF, including the rfABF approach used in this study, exclusively operates by reallocating acoustic frequencies to electrode contacts; the physical position of electrode contacts cannot be altered by any fitting strategy. Interaural mismatch is therefore expressed in degrees as a metric of interaural place-of-stimulation mismatch at matched acoustic frequency locations. Accordingly, the reported reduction reflects a decrease in interaural place-of-stimulation mismatch resulting from frequency reallocation to existing electrode contacts, without any change in electrode contact position or insertion depth.

### 3.4 Frequency dependence of interaural place mismatch

To quantify the effect of rfABF across the frequency range, a linear mixed-effects model was applied with signed interaural place mismatch as the dependent variable, a random intercept for participant and fixed factors for fitting method (CBF vs. rfABF), log-transformed reference-side filter-band center frequency, and their interaction. The model confirmed that rfABF significantly reduced interaural mismatch (Satterthwaite ANOVA: (F (1,183.98)=5.80, p=0.017; Fig 2). Neither the main effect of frequency nor the Method x Frequency interaction were significant (frequency: F(1,184.33)=2.47, p=0.118; interaction F(1,184.27)=2.12, p=0.147).

**Fig 2:**
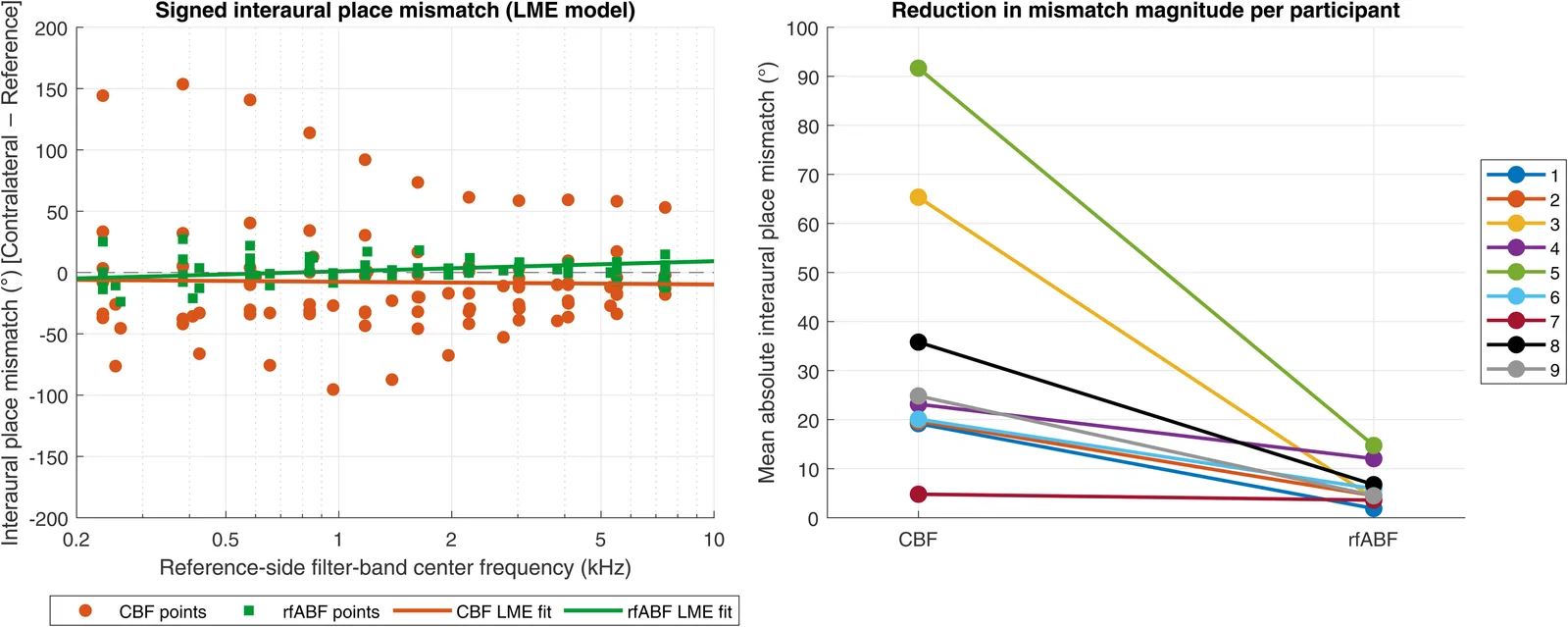
Interaural place mismatch and reduction with refined anatomy-based fitting. **A:** Signed interaural place mismatch (Contralateral – Reference, °) as a function of reference-side filter-band center frequency (kHz). Red circles show observations with the CBF map and green squares show observations with the rfABF map (pooled across participants). Solid lines are linear mixed-effects (LME) model predictions for each condition (CBF: red, rfABF: green) The x-axis is logarithmic (0.2-10 kHz); the horizontal dashed line marks zero mismatch. **B**: Mean absolute interaural place mismatch per participant for CBF and rfABF, illustrating the reduction in mismatch magnitude with rfABF across participants. Each line represents an individual participant (color-coded by subject ID). CBF = clinically based fitting; rfABF = refined anatomy-based fitting.

As illustrated in Fig 2A (left panel), signed interaural place mismatch values for CBF spanned a wide range of positive and negative values across frequency, reflecting substantial interaural place mismatch with both apical and basal deviations, whereas rfABF resulted in a markedly tighter clustering of signed mismatch values around zero across the frequency range.

Across participants, mean absolute interaural place mismatch decreased from 33.8 ± 27.3∘ with CBF to 6.4 ± 4.2∘ with rfABF mapping, representing an 81% reduction. The per-participant reduction in mismatch magnitude is shown in Fig 2B (right panel). The within-participant reduction was significant (paired t(8)=3.25, p=0.0112; 95% CI for the mean difference 8.1° - 46.7°). The effect size was large (Cohen’s dz=1.09). Given the small sample size, a Wilcoxon signed-rank test was additionally performed as a sensitivity analysis to assess the robustness of the paired comparison; this non-parametric test yielded consistent results (p = 0.0039).

### 3.5 Refined (rfABF) vs. calculated ABF (${ABF}_{calculated})$

To evaluate how closely the purely algorithm-derived anatomy-based frequency allocations obtained with zipped ABF or transposed ABF (collectively, ${ABF}_{calculated}$) aligned with the final rfABF map, we compare the calculated ABF maps with the corresponding rfABF maps for each participant. ${ABF}_{calculated}$ represents the direct output of the selected algorithm prior to any participant-guided psychoacoustic adjustment, whereas rfABF reflects the final map after the refinement step, as implemented within the fitting software.

Across all 9 participants, only small deviations between ${ABF}_{calculated}$ and rfABF were observed (Fig 3). These deviations do not indicate intentional frequency remapping during refinement but arise from the implementation of frequency bands within the fitting software, which determines how algorithm-derived frequency boundaries are mapped onto discrete electrode channels. In the middle (200°–400°) and apical (>400°) regions, these deviations were minimal and clinically negligible, with pooled mean absolute differences of 13.7 ± 11.3 Hz (middle) and 9.8 ± 9.4 Hz (apical). In contrast, larger differences were observed in the basal (0–200°; 172.2 ± 274.5 Hz), consistent with greater anatomical variability and reduced frequency resolution in the basal cochlea.

**Fig 3:**
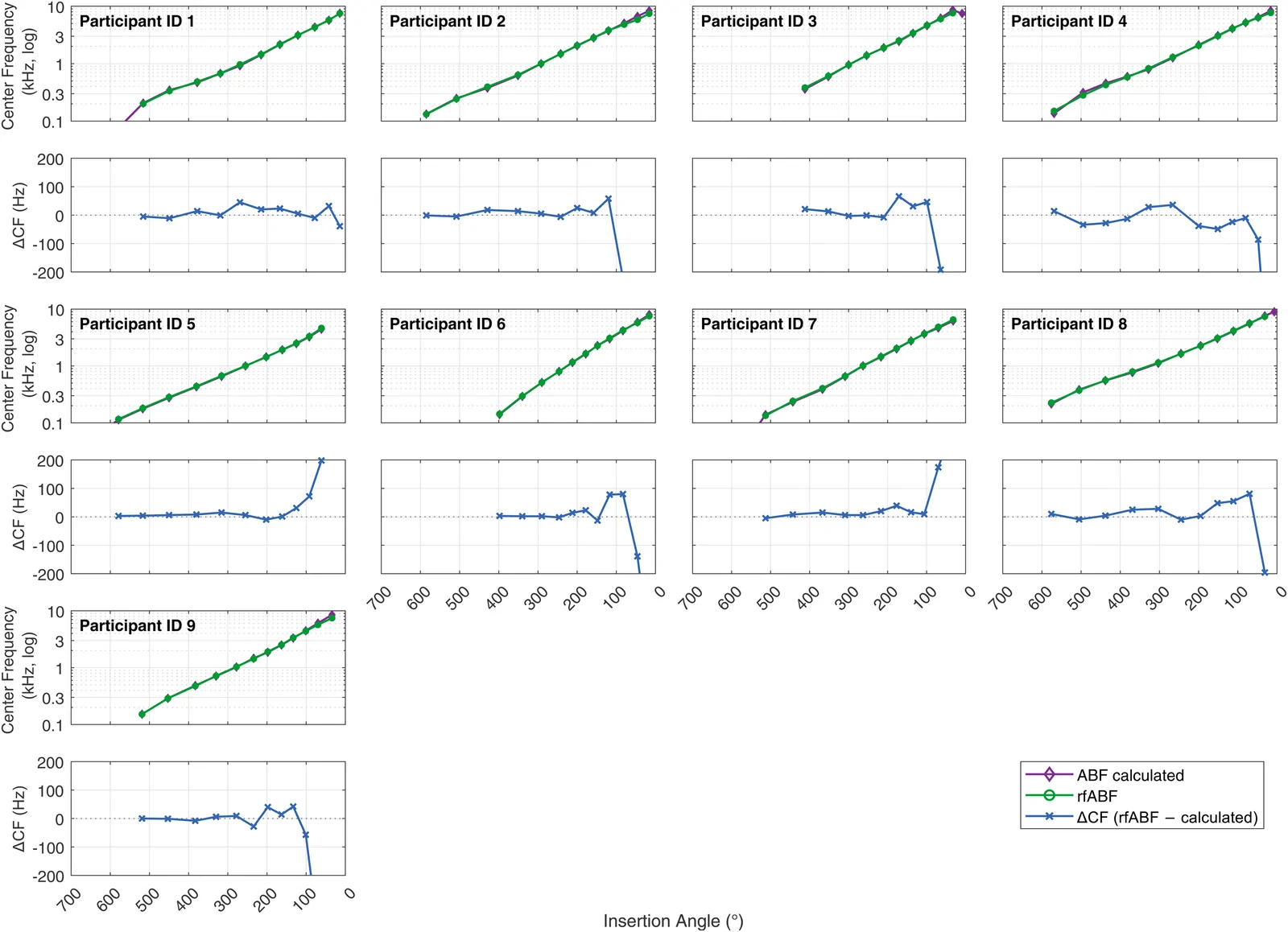
Individual comparison between the algorithm-derived anatomy-based fitting map (ABFcalculated)—generated using either zipped ABF or transposed ABF depending on participant preference—and the final refined anatomy-based fitting map (rfABF) after psychoacoustic fine-tuning. For each participant, the upper row shows the relationship between the insertion angle (°) and filter-band center frequency (kHz, logarithmic scale) for ABFcalculated (purple line, diamond data points) and rfABF (green line, circle data points). The lower row shows the absolute frequency differences between rfABF and ABFcalculated (ΔCF = rfABF-ABFcalculated, in Hz) as a function of insertion angle.

Overall, these findings indicate that rfABF preserves the tonotopic relationship defined by the selected ABF algorithm, while incorporating limited, participant-specific loudness (MCL) adjustments during the refinement process.

### 3.6 Speech perception performance

#### 3.6.1 Speech understanding in quiet.

Individual results for speech comprehension in quiet and in noise can be found in the supplementary material (see S1 and S2 Tables Supplemental Digital Content).

The improvement in the bilateral setup was statistically significant in favor of the rfABF map (t = −2.73 df = 8; p = 0.025; Fig 4A). No significant differences between the CBF map and the rfABF map were found for the reference ear (t = −0.667; df = 8; p = 0.524) or the contralateral ear (t = −0.529; df = 8; p = 0.529) when tested alone.

**Fig 4:**
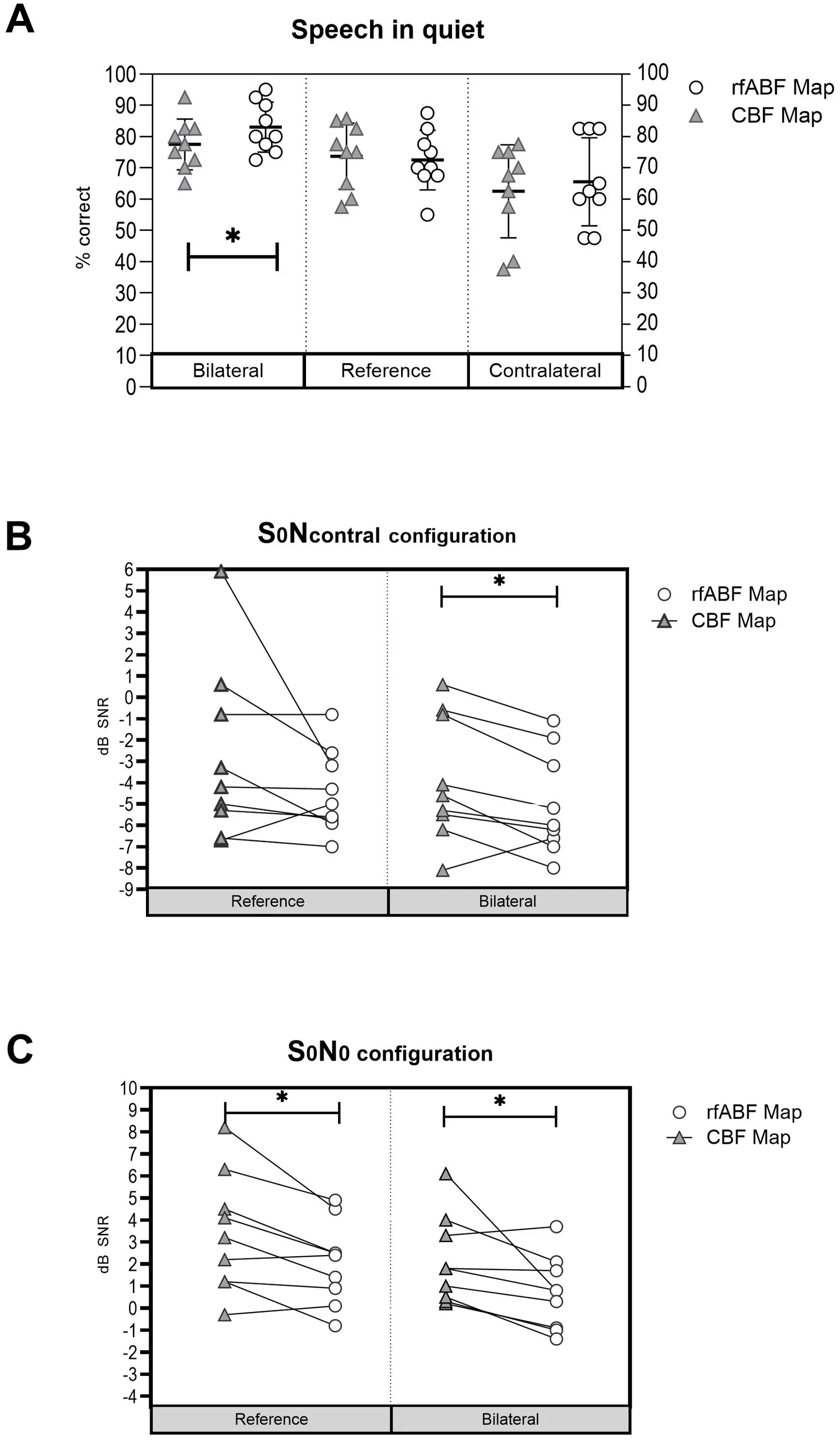
Speech comprehension in quiet and in noise. A. Speech understanding in quiet assessed using the German Freiburg Monosyllables test at 65 dB SPL in 3 listening conditions: bilateral, reference ear only, and contralateral ear only. Data points show scores for each participant in each listening condition. **B, C**. Speech understanding in noise using the Oldenburg Sentence Test (OLSA) test with a variable masker level and a constant speech level of 65 dB SPL in 2 spatial configurations: S0Ncontra (B) and S0N0 (C), and in 2 listening conditions: bilateral and reference ear only. Data points show scores for each participant in each listening condition. CBF = clinically based fitting; rfABF = refined anatomy-based fitting.

#### 3.6.2 Speech understanding in noise.

Speech-in-noise results compare performance at baseline and at follow-up after 4 weeks of rfABF use. Because the rfABF map was applied exclusively to the contralateral ear, results obtained in the reference ear reflect baseline-to-follow-up differences under the rfABF map and therefore represent visit-to-visit variability rather than map-specific effects.

##### 3.6.2.1 S0N_contra_ configuration:

In the bilateral listening condition, the mean SRT score was significantly better at follow-up with the rfABF map than at baseline with the CBF map (-5.0 dB ± 2.4 dB vs −3.84 dB ± 2.9 dB; t = 2.974; df = 8; p = 0.0178; Fig 4B).

In the reference ear, the mean SRT score at follow-up was lower than at baseline, although this did not reach significance (−4.5 dB ± 1.9 dB vs. −2.8 dB ± 4.1 dB; t = 1.555; df = 8; p = 0.158).

##### 3.6.2.2 S_0_N_0_ configuration:

In the bilateral listening condition, the mean SRT score was significantly better at follow-up with the rfABF map than at baseline with the CBF map (0.7 dB ± 1.7 dB vs. 2.1 dB ± 2.0 dB; t = 2.63; df = 8; p = 0.03; Fig 4C).In the reference ear, the mean SRT score at follow-up was significantly better than at baseline (2.0 dB ± 1.9 dB vs. 3.4 dB ± 2.7 dB; t = 3.166; df = 8; p = 0.013).

### 3.7 Localization accuracy and sound quality

#### 3.7.1 Localization accuracy.

Localization accuracy (RMS error) ranged from 13.3° to 35.0° with the CBF map and from 10.30° to 25.7° with the rfABF map (participant estimates and bootstrap 95% CIs in Table 2). Group-level mean RMS error decreased from 22.40° with CBF to 20.61° with rfABF (N=7; 2 participants did not complete localization), corresponding to a mean change of −1.79° (Table 3).

**Table 2:** Localization accuracy (RMS error and signed bias) and self-rated sound quality (HISQUI_19_) for the clinically based fitting map (CBF) and the refined anatomy-based map (rfABF).

ID	Localization												HISQUI_19_	
	RMS (°)						BIAS (°)						CBF	rfABF
	CBF			rfABF			CBF			rfABF				
	Mean	95% CI		Mean	95% CI		Mean	95% CI		Mean	95% CI			
		LL	UL		LL	UL		LL	UL		LL	UL		
1	NA	NA	NA	NA	NA	NA	NA	NA	NA	NA	NA	NA	109	112
2	13.3	11.99	14.58	10.3	8.66	11.86	−1.8	−3.19	0.42	1.7	0.42	2.92	88	88
3	19.8	17.68	21.65	21.3	19.36	23.12	7.9	5.56	10.28	4.9	2.64	7.08	47	48
4	24	21.94	25.98	25.6	23.05	28.01	4.4	2.22	6.53	−2.1	−4.86	0.69	109	102
5	23.5	21.29	25.56	18.6	16.11	21.21	−1.4	−3.47	0.56	−0.1	−1.94	1.81	62	76
6	18.2	15.71	20.69	17.8	15.81	19.69	1.7	−0.69	3.89	1.8	−0.14	3.89	51	49
7	23	20.92	25.19	25	22.78	27.10	−0.8	−3.19	1.53	−6.9	−9.31	−4.58	60	61
8	35	31.82	38.04	25.7	22.50	28.94	2.5	−1.53	6.39	0	−3.19	3.19	59	65
9	NA	NA	NA	NA	NA	NA	NA	NA	NA	NA	NA	NA	93	96

*Note.* RMS = root-mean-square localization error (°). Bias = signed mean localization error (°; positive = responses toward larger loudspeaker angles). NA indicates missing/not obtained data. Localization metrics (RMS error and signed bias) are reported only for participants who completed the localization task (N=7), and corresponding summary statistics are computed across these participants. HISQUI_19_ scores are available for all participants (N=9), and summary statistics for HISQUI_19_ include all participants. Higher HISQUI_19_ scores reflect better self-reported hearing quality. CBF = clinically based fitting; rfABF = refined anatomy-based fitting; LL = lower limit; UL = upper limit.

**Table 3:** Mean localization accuracy (RMS error and signed bias) for all participants under the clinically based map (CBF) and the refined anatomy-based map (rfABF). Values are means with bootstrap-derived 95% percentile intervals (2.5–97.5) in degrees.

Metric	CBF			rfABF			Δ = rfABF–CBF		
	Mean	95% CI		Mean	95% CI				
							Mean	95% CI	
		LL	UL		LL	UL		LL	UL
RMS error (°)	22.40	18.09	27.33	20.61	16.38	23.98	–1.79°	–4.90	+0.81
Signed bias (°)	1.79	−0.50	4.29	–0.12	−2.77	2.20	–1.9°	–4.42	+0.65
HISQUI_19_ (pts)	75.3	49	110	77.4	52	113	+1.9 pts	–2.3	+6.7

*Note.* RMS = root-mean-square localization error (°). Bias = signed mean localization error (°; positive = responses toward larger loudspeaker angles). CBF = clinically based fitting; rfABF = refined anatomy-based fitting; LL = lower limit; UL = upper limit.

Signed localization bias showed a similar pattern. Mean signed bias shifted from 1.79∘ with CBF toward −0.12∘ with rfABF, yielding a mean change of −1.90° (Table 3). At the individual level, bias values were reduced in magnitude with rfABF compared to CBF in 5 of the 7 tested participants (Table 2).

Bootstrap-derived confidence intervals for group-level changes in RMS error and signed bias are reported in Table 3.

#### 3.7.2 HISQUI_19_.

Self-rated sound quality (HISQUI_19_) showed no disadvantage for rfABF and a trend toward higher scores (per-participant values are reported in Table 2). Six of 9 participants (67%) reported higher HISQUI_19_ scores with the rfABF map than with CBF; 2 participants (22%) had lower scores; 1 (11%) was unchanged.

Using the standard HISQUI_19_ categories, CBF yielded 1 “very good”, 2 “good”, 4 “moderate”, and 2 “poor” whereas rfABF yielded 3 “good”, 4 “moderate”, and 2 “poor”. Group means (± SD) were 75.3 ± 24.5 for CBF and 77.4 ± 23.3 for rfABF; the mean difference was not statistically significant, t = 1.101, df = 8, p = 0.303, but the direction favored rfABF (Table 3).

### 3.8 Listening effort

The pupillometry analysis was exploratory and descriptive. Because of the small number of participants and the inter-individual variability characteristic of pupil responses, data are presented at the individual level rather than tested for group-level significance.

Pupillometry data were available for 8 participants (Fig 5); 1 participant (ID 5) did not complete the assessment and was therefore excluded from this analysis. Across participants, pupil-response patterns differed between CBF and rfABF. In 4 participants (IDs 3, 4, 6, and 8) the dilation traces with rfABF were consistently lower than with CBF. Speech performance in these cases with rfABF was equal to or higher than with CBF. The average reduction in peak/mean %ERPD for these participants was −2.47 percentage points (range −6.54 to −0.46), accompanied by changes in OLSA sentences of +2.7 percentage points on average (range −1.3 to 8.0).

**Fig 5:**
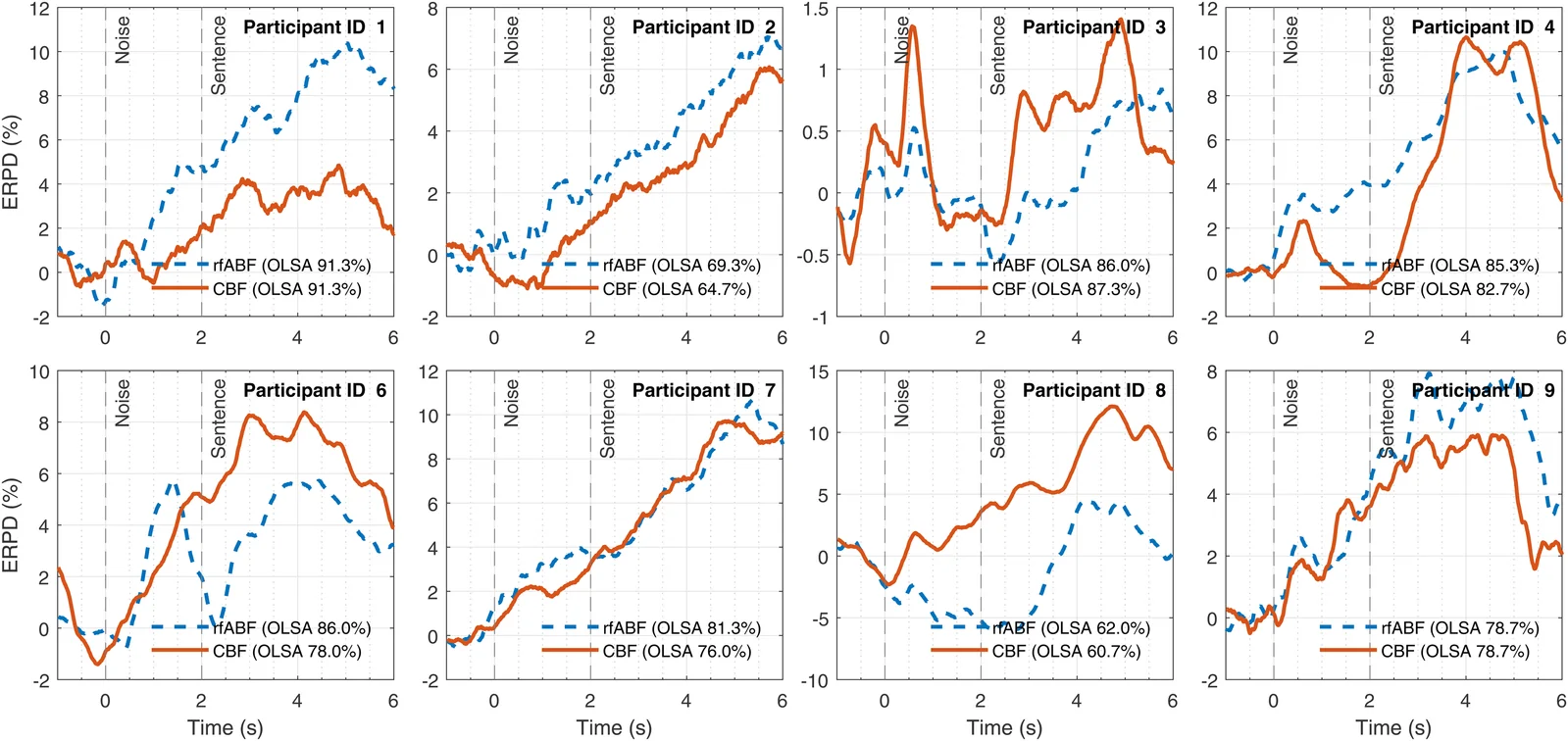
Pupillary responses (expressed as Event-Related Pupil Dilation percentage, ERPD%) during the OLSA speech in noise task comparing the CBF map (solid red line) and rfABF map (dashed blue line). Vertical dashed lines indicate noise onset (0 s) and speech target onset (2 s). CBF = clinically based fitting; rfABF = refined anatomy-based fitting.

One participant (ID 7) showed comparable pupil responses across fittings while speech performance improved from 78% (CBF) to 86% (rfABF).

Less favorable patterns were limited. One participant (ID 2) exhibited higher pupil dilation with rfABF, accompanied by a reduction of approximately 5 percentage points in OLSA performance. In 2 participants (IDs 1 and 9), speech performance was similar across maps but rfABF required more effort in both cases, with a smaller difference for participant 9.

## 4 Discussion

The aim of this study was to evaluate a method for reducing interaural place mismatch in bilateral cochlear implant (CI) users by refining the concept of anatomy-based fitting (ABF). Rather than minimizing mismatch between the CI frequency allocation and the cochlear tonotopic map within a single ear, the present work specifically targets bilateral mismatch between the 2 implanted sides, which is known to limit access to binaural cues [31]. This was achieved through 2 approaches—zipped ABF and transposed ABF—combined with participant-guided psychoacoustic fine-tuning. In contrast to conventional clinically based fitting (CBF), both approaches reallocate cochlear frequency-band center frequencies according to individual electrode array insertion angles from each ear. This underscores the need for imaging analysis and the use of surgical planning software such as OTOPLAN.

When comparing the algorithm-derived ABF maps (${ABF}_{calculated}$) with the final refined anatomy-based fitting rfABF maps for each participant, the calculated ABF maps closely matched the final rfABF maps. Differences between ${ABF}_{calculated}$ and rfABF were minimal in the middle and apical cochlear regions, but substantially larger in the basal region. These differences do not arise from psychoacoustic fine-tuning but reflect the implementation of frequency bands within the fitting software. Accordingly, while the calculated ABF map appears sufficiently accurate for direct clinical application in the middle and apical regions, limited refinement may be required in the basal regions. Overall, these results affirm the robustness and accuracy of the calculated ABF method and its suitability as a solid base for personalized clinical fitting.

A linear mixed-effects model was used to assess the impact of the rfABF method on interaural place mismatch. The model confirmed that rfABF significantly reduced interaural mismatch. In addition, mean absolute interaural place mismatch was substantially reduced with rfABF, reaching values within ≤10∘, which falls well within the 75° tolerance range previously associated with effective binaural-cue processing (Bernstein et al., 2021). Together, these findings indicate that rfABF achieves close bilateral alignment of place-of-stimulation across ears, which may facilitate binaural auditory processing.

rfABF substantially improved speech understanding in both quiet and noisy environments under bilateral listening conditions. These findings build upon previous work [33], which indicated the need for tailored fitting solutions for CI users with uneven bilateral electrode array lengths. That earlier study showed that ABF improved speech perception in noisy environments for CI users with complete cochlear coverage on one side, once frequency bands were removed on the contralateral ear in order to correctly stimulate tonotopically. The present study extends this concept by showing that rfABF can equalize bilateral place-of-stimulation alignment, potentially facilitating access to binaural cues. Not all studies have reported speech perception benefits from anatomy-based fitting. Lassaletta et al. [20] found no significant group-level differences in speech scores between ABF and default fitting in experienced CI users, although a majority of participants preferred the ABF map, and image-guided fitting approaches across different electrode platforms and patient cohorts have yielded variable or mixed speech outcomes [18,21]. Recent work also suggests that the estimated magnitude of tonotopic mismatch may be less extreme than traditionally assumed when conversational stimulation levels are accounted for [17], while behavioral evidence indicates that CI users retain considerable central auditory plasticity to adapt to shifted frequency allocations over time [32]. The present findings should therefore be interpreted within a broader literature in which the clinical benefit of place-based mapping remains variable across listener populations, adaptation durations, and outcome measures.

Although localization accuracy, which depends in part on binaural cue processing, did not show a significant improvement, the slight reduction in RMS error after 4 weeks of using the rfABF map suggests that rfABF does not compromise spatial hearing compared to CBF mapping. A larger sample size and/or a repetition of the task may be necessary to detect more robust effects. Importantly, our study found significantly improved speech perception scores in both quiet and noise in the bilateral condition, consistent with improved access to binaural cues.

Self-reported overall HISQUI_19_ scores did not significantly increase with rfABF, but they did increase by either 1 or 2 points in 6 participants and remained stable in 3 participants. This suggests that rfABF may reduce the perceivable spectral distortion, thereby contributing to a more natural listening experience. Patient-reported outcomes such as these are essential in evaluating the real-world impact of CI fitting strategies because they reflect the user’s perception of sound quality and everyday listening experiences.

Pupillometry provided complementary evidence on listening effort when comparing rfABF with CBF. A majority of participants showed lower event-related pupil dilation (ERPD) with rfABF when speech understanding was equal or improved, indicating reduced listening effort during sentence-in-noise processing. One additional participant exhibited improved intelligibility with comparable pupil responses across maps, suggesting that speech benefits can emerge even when listening effort remains stable. Less favorable patterns were observed in a smaller number of participants, underscoring inter-individual variability in effort responses. These observations are consistent with previous pupillometry findings in bilateral CI users showing that interaural asymmetry can increase listening effort, with bilateral listening sometimes proving more effortful than better-ear listening alone [37].

These findings are consistent with previous reports [46] showing that pupil responses tend to compress at higher intelligibility levels, leading to smaller between-condition ERPD differences when tasks become easier. This pattern reflects the well-documented non-monotonic relationship between task difficulty and pupil dilation, whereby listeners may disengage at very high difficulty levels, producing reduced rather than increased dilation [43]. Within this framework, lower ERPD observed alongside equal or better speech performance can be interpreted as reduced listening effort for comparable accuracy. By contrast, unchanged or increased ERPD suggests that some listeners require additional processing effort despite similar performance [35]. Accordingly, the present pupillometry findings provide complementary evidence that rfABF can be associated with reduced listening effort in some bilateral CI users. Because both maps were tested within the same session at an identical SNR, the observed differences in event-related pupil dilation cannot be attributed to differences in acoustic signal-to-noise ratio between conditions. Nevertheless, interpretable patterns emerged in only a subset of participants, so these pupillometry results remain exploratory and should be regarded as hypothesis-generating rather than confirmatory.

From a clinical perspective, listening effort may relate to everyday comfort. While longer observation would be needed to assess durability and function impact, pairing speech measures with effort indices can provide a more complete picture of listener experience, complementing patient-reported tools such as HISQUI_19_.

Several limitations should be considered. First, the sample size was small (n = 9), reflecting the restricted population of bilateral CI users with incomplete cochlear coverage and asymmetric insertion. For a number of years, our clinic has adopted a strategy of delivering the highest standard of care to all CI recipients, with complete cochlear coverage being a central component of this approach. Consequently, it proved challenging to recruit patients for this study. It was therefore not possible to establish a control group. Because the reference ear, whose frequency map was not modified, also showed improvement at follow-up, test-retest and acclimatisation effects cannot be excluded, and improvements observed at the bilateral level cannot therefore be attributed to the rfABF map alone. As the sensitivity analysis indicates, the study was powered to detect only large within-subject effects ($d_{z}\approx \text{1}.\text{0}\text{7}$). This limits the interpretation of the non-significant comparisons for unilateral speech, localization and HISQUI_19_, which should be regarded as inconclusive rather than as evidence of no effect. The significant improvements in interaural mismatch and bilateral speech understanding were obtained despite this constraint and are therefore unlikely to reflect insufficient sensitivity; however, effect sizes estimated from small samples tend to be upwardly biased, so the magnitude of these benefits requires confirmation in a larger cohort. Second, a large number of outcome measures were assessed relative to the sample size; although Holm-Bonferroni correction was applied within each outcome family, the risk of Type I error across the full set of analyses cannot be excluded, and all findings should be regarded as preliminary and hypothesis-generating. Third, while 4 weeks may be sufficient for initial adaptation, it may not fully capture long-term effects of rfABF on auditory performance, sound quality perception, or listening effort. Extended follow-up could reveal whether the benefits observed are sustained or evolving over time.

Overall, these findings contribute to the ongoing development of tailored CI fitting strategies that prioritize not only speech understanding but also listening ease and sound quality.

## 5 Conclusion

This study uses a refined anatomy-based fitting (rfABF) method, designed to individualize frequency allocation and reduce interaural place-of-stimulation mismatch in bilateral CI users. Developed specifically for cases with incomplete cochlear coverage, rfABF significantly improved speech understanding in both quiet and complex listening environments. Beyond auditory performance, rfABF was associated with preliminary evidence for reduced listening effort and stable or improved perceived sound quality. These proof-of-concept findings indicate the importance of anatomically guided, personalized strategies and support further evaluation of rfABF in larger cohorts [28,47].

## Supporting information

S1 Fig Test setup. Auditory stimuli for speech testing were presented from loudspeakers positioned at −90°, 0°, and +90° azimuth (1, 5, and 9). In an additional speech-in-noise test for pupillometry, participants were asked to shift slightly to the left to face a computer screen positioned precisely between loudspeakers 3 and 4. For this task auditory stimuli were presented at −11.25° and +11.25° azimuth relative to the participant.
(DOCX)

S1 Table Speech understanding in quiet. Individual speech scores and mean (±SD standard deviation) in percent correct (%) measured using Freiburg Monosyllables at 65 dB SPL with the clinically based fitted (CBF) and the refined anatomy-based fitted (rfABF) map for each participant. Bilateral, Reference Side, and Contralateral Side refer to the measured condition using both ears or individual ears.
(DOCX)

S2 Table Speech understanding in noise for each participant at each setup and each map. Mean (±SD standard deviation) speech understanding in noise in the S0N0 test condition at each setup and each map with the clinical-based fitted (CBF) and the refined anatomy-based fitted (rfABF) map. Scores are given as signal-to-noise (SNR) ratio required for 50% correct understanding of words; thus, lower scores indicate better results.
(DOCX)

## Abbreviations

ABF: anatomy-based fitting
AID: angular insertion-depth
CBF: clinically based fitting
CCITT: speech-shaped noise defined by the Comité Consultatif International Teléphonique et Télégraphique
CI: cochlear implant
CT: computed tomography
ERPD: event-related pupil dilation
fpVCT: flat panel volume computed tomography
FSP: fine structure processing
FS4/FS4-p: fine structure processing strategies (4 fine-structure channels / parallel variant)
HISQUI19: hearing implant sound quality index
HRTF: head-related transfer function
ILD: interaural level difference
ITD: interaural time difference
LME: linear mixed-effects
MCL: most comfortable levels
OLSA: oldenburg sentence test
rfABF: refined anatomy-based fitting
RMS: root-mean square
SD: standard deviation
SNR: signal-to-noise ratio
SPL: sound pressure level
SRT: speech reception threshold
