# Supplementary material for: Refined anatomy-based fitting reduces interaural place mismatch and improves speech outcomes, with preliminary evidence for reduced listening effort
Source: PLoS One. 2026 Oct 26;21(10):e0361044. doi: 10.1371/journal.pone.0361044 (PMC13704877; doi:10.1371/journal.pone.0361044)
Supplement: Supplementary file 1 [file pone.0361044.s001.docx]

Figure S1 Test setup. Auditory stimuli for speech testing were presented from loudspeakers positioned at -90°, 0°, and +90° azimuth (1, 5, and 9). In an additional speech-in-noise test for pupillometry, participants were asked to shift slightly to the left to face a computer screen positioned precisely between loudspeakers 3 and 4. For this task auditory stimuli were presented at -11.25° and +11.25° azimuth relative to the participant.
