# Supplementary material for: Refined anatomy-based fitting reduces interaural place mismatch and improves speech outcomes, with preliminary evidence for reduced listening effort
Source: PLoS One. 2026 Oct 26;21(10):e0361044. doi: 10.1371/journal.pone.0361044 (PMC13704877; doi:10.1371/journal.pone.0361044)
Supplement: Supplementary file 2 [file pone.0361044.s002.docx]

Table S1. Speech understanding in quiet. Individual speech scores and mean (±SD standard deviation) in percent correct (%) measured using Freiburg Monosyllables at 65 dB SPL with the clinically based fitted (CBF) and the refined anatomy-based fitted (rfABF) map for each participant. Bilateral, Reference Side, and Contralateral Side refer to the measured condition using both ears or individual ears.

| ID | Speech in quiet (% correct) | | | | | |
| --- | --- | --- | --- | --- | --- | --- |
|  | Bilateral | | Reference Side | | Contralateral Side | |
|  | CBF Map | rfABF Map | CBF Map | rfABF Map | CBF Map | rfABF Map |
| 1 | 80.0 | 95.0 | 85.0 | 87.5 | 75.0 | 82.5 |
| 2 | 82.5 | 80.0 | 82.5 | 75.0 | 77.5 | 60.0 |
| 3 | 72.5 | 77.5 | 75.0 | 70.0 | 62.5 | 47.5 |
| 4 | 92.5 | 92.5 | 85.7 | 82.5 | 70.0 | 82.5 |
| 5 | 75.0 | 85.0 | 65.0 | 70.0 | 37.5 | 65.0 |
| 6 | 77.5 | 75.0 | 75.0 | 67.5 | 67.5 | 62.5 |
| 7 | 65.0 | 72.5 | 57.5 | 55.0 | 40.0 | 47.5 |
| 8 | 70.0 | 80.0 | 60.0 | 67.5 | 57.5 | 60.0 |
| 9 | 82.5 | 90.0 | 77.5 | 77.5 | 75.0 | 82.5 |
| Mean total | 77.5 | 83.1 | 73.7 | 72.5 | 62.5 | 65.6 |
| SD | 8.1 | 8.0 | 10.6 | 9.5 | 14.9 | 14.1 |
