# Supplementary material for: Refined anatomy-based fitting reduces interaural place mismatch and improves speech outcomes, with preliminary evidence for reduced listening effort
Source: PLoS One. 2026 Oct 26;21(10):e0361044. doi: 10.1371/journal.pone.0361044 (PMC13704877; doi:10.1371/journal.pone.0361044)
Supplement: Supplementary file 3 [file pone.0361044.s003.docx]

Table S2. Speech understanding in noise for each participant at each setup and each map. Mean (±SD standard deviation) speech understanding in noise in the S0N0 test condition at each setup and each map with the clinical-based fitted (CBF) and the refined anatomy-based fitted (rfABF) map. Scores are given as signal-to-noise (SNR) ratio required for 50% correct understanding of words; thus, lower scores indicate better results.

| ID | Speech in noise (S0N_contralateral_) | | | | Speech in noise (S0N0) | | | |
| --- | --- | --- | --- | --- | --- | --- | --- | --- |
|  | Reference side  only | | Bilateral | | Reference side  only | | Bilateral | |
|  | CBF Map | rfABF Map | CBF Map | rfABF Map | CBF Map | rfABF Map | CBF  Map | rfABF Map |
| 1 | -6.7 | -5 | -8.1 | -6.6 | -0.3 | 0.1 | 0.2 | -0.9 |
| 2 | -5 | -5.7 | -4.6 | -7 | 4.5 | 2.5 | 4 | 2.1 |
| 3 | -3.3 | -5.9 | -4.1 | -5.2 | 3.2 | 1.4 | 1.8 | 0.8 |
| 4 | -5.3 | -5.6 | -5.5 | -6.2 | 1.2 | 0.9 | 0.3 | -1 |
| 5 | -4.2 | -4.3 | -5.3 | -6 | 2.2 | 2.4 | 1.8 | 1.7 |
| 6 | -6.6 | -7 | -6.2 | -8 | 1.2 | -0.8 | 0.5 | -1.4 |
| 7 | 5.9 | -3.2 | 0.6 | -1.1 | 8.2 | 4.5 | 6.1 | 0.8 |
| 8 | -0.8 | -0.8 | -0.6 | -1.9 | 6.3 | 4.9 | 3.3 | 3.7 |
| 9 | 0.6 | -2.6 | -0.8 | -3.2 | 4.1 | 2.5 | 1 | 0.3 |
| Mean | -2.8 | -4.5 | -3.8 | -5 | 3.4 | 2 | 2.1 | 0.7 |
| SD | 4.1 | 1.9 | 2.9 | 2.4 | 2.7 | 1.8 | 2 | 1.6 |
